# A Genealogical Study of Facemasks in China: From Hygienic Modernity to Care

**DOI:** 10.1007/s40647-022-00343-z

**Published:** 2022-02-07

**Authors:** Buyun Gong

**Affiliations:** Institute for Interdisziplinäre Anthropologie, Albert-Ludwigs-Universität Freiburg, Freiburg Im Breisgau, Germany

**Keywords:** Facemask, Biopolitics, Care, Subjectification, SARS, Manchurian plague

## Abstract

Despite the omnipresence of facemasks in the ongoing COVID-19 pandemic, recent studies on their sociopolitical aspects remain insufficient. This article conducts a genealogical study that investigates the emergence of two differing masking strategies in two epidemic events in Chinese history. First, during the Manchurian plague 1910/11, it shows how the germ theory and historical anecdotes made anti-plague masks thinkable and practicable as a solution not only for airborne contagion but also for the biopolitical problem of ‘unhygienic’ population. In the second part, the analytical focus is shifted to the emergence of collective mask-wearing practices during SARS 2002/03 from the vantage point of subjectification. Facemasks then became a symbol of care in the neoliberal regime of responsibilization. This article concludes by arguing for a rethinking of facemasks as actants who actively participate in the constitution of the world we share.

## Introduction

If there is one object that symbolizes and crystalizes our collective experiences during the Covid-19 pandemic, it is, without doubt, the facemask. It is both a central component of the administratively prescribed strategies to contain the pandemic and—in addition to social distancing—the most widespread personal protective measure. That being said, the strategy of mass masking was not self-evident at the beginning of the Covid-19 outbreak. The act of mask-wearing was first perceived as a unique East Asian phenomenon as Covid-19 started to gain global media attention. Medical authorities outside of East Asia, including the WHO, were generally reluctant to impose mass masking in public. However, in early April 2020, an increasing number of governments in Western countries changed their facemask policy and started to issue guidelines for universal mask-wearing (Goldstein et al. 2020). The use of facemasks by the general population was framed as an urgent and effective *strategy* to impede COVID-19 transmission (Gandhi et al. 2020; Howard et al. 2021; Holakouie-Naieni et al. 2020).

Facemasks in times of COVID-19 are more than just biomedical devices covering one’s mouth and nose. The global spread of the virus has made them highly charged symbols in political debates, popular culture, and everyday interactions. In order to make sense of their dynamic symbolic meanings, an increasing number of social and cultural studies on masks have been published since the outbreak of COVID-19. Studying the socio-material meanings of COVID facemasks, Lupton et al. (2021: 84) reveal “the manifold complexities, forces and intra-actions of symbolism, discourse, politics, culture, and embodiment in which the face mask in COVID is entangled.” Ma and Zhan (2020) make visible social stigmatization experienced by masked Chinese students in America. The so-called ‘mask culture’ is criticised by Zhang (2021) as an Orientalist discourse that perpetuates and reproduces stereotypes about Asian people. Last but not least, Han (2020: 6) reveals how Chinese official facemask narratives have constructed mask-wearing "as a deontic value that all citizens should embrace”.

This article, however, does not engage directly with the present-day phenomena of masking, but rather its ‘history of the present’ (Roth 1981; Dean 1994; Garland 2014). From a genealogical perspective, meanings are historically contingent and are only fixated through the operation of power (Foucault 1984). It is therefore important to pay attention to the process of meaning-making that is located in specific historical contexts. To be more specific, this article traces the genealogy of masking in the history of modern China. Genealogy is, however, different from history. A conventional history of facemasks may simply juxtapose different designs of masks from different periods in a temporally linear and continual order. This, however, often runs the risk of technological determinism. Genealogists, on the other hand, focus on discontinuities and multiple processes of emergence. It is a reflexive art of problematization that traces how power struggles and contingency are constitutive of the emergence of contemporary practices and institutions (Garland 2014: 372). In this sense, a genealogical study of masks does not intend to formulate a sweeping social theory of masking, but to develop some heuristic tools that make sense of and problematize present-day masking practices.

In what follows, this article focuses on socio-material conditions that give rise to the emergence of masking strategies in the Chinese history of epidemic control. Schematically speaking, it examines two kinds of masking strategies: One that is implemented in a top-down manner and one that is initiated by the public ‘from below’. These two strategies emerge in two epidemic events. The first one concerns the birth of modern facemasks (gauze masks) in the context of the Manchurian plague 1910/11. The anti-plague mask was first made thinkable by new forms of medical knowledge and historical anecdotes, then implemented as a biopolitical strategy aiming at an ‘unhygienic’ population, and finally celebrated as a symbol of “hygienic modernity” (Rogaski 2004). The second moment witnessed the emergence of collective mask-wearing practices in the context of SARS 2002/03. The central question this time was not so much a medico-political one concerning *implementing* masking policy, but rather an ethical question on *wearing* them. How are people incited to recognize their obligations to wear facemasks? During the SARS outbreak, facemasks became a symbol of care in the neoliberal regime of responsibilization. In the last section, this article will discuss how this genealogical study of masks can contribute to a rethinking in COVID times.

## The Emergence of Anti-plague Masks

At the end of 1910, a year before the collapse of the Qing dynasty, a vehement plague ravaged Northern Manchuria. By the time the epidemic had waned in March 1911, it had cost more than 60,000 lives in total. The fatality rate proved to be 100%. The plague first made its appearance in Manzhouli, a Chinese town bordering on Russia. Along the railway lines and roads, the disease surged southwards to other cities in Manchuria—a vast territory contested by Japan, Russia, and Qing. At the outset of the outbreak, the Russians were quicker to react to the plague than the Qing government. The disease was first diagnosed in the Russian concession city of Harbin on 27 October and an improvised plague hospital was soon built (Gamsa 2006: 148). At the same time, Japan was putting diplomatic pressure on Beijing while preparing to invade Manchuria from Korea. Both countries questioned Qing’s ability to contain the plague and framed the plague epidemic as a political issue of international importance (Flohr 1996: 367; Bu 2017: 48). Unlike previous plague events, which were ‘perceived’ by Qing as a regional or national issue, the outbreak this time posed a threat to Qing’s sovereignty over Manchuria (Lei 2010: 77). Instead of resorting to war and the army, which was a common solution to sovereignty problems, the outbreak of the Manchurian plague required the Qing government to resort to new strategies.

In the midst of this crisis, China’s vice minister of foreign affairs, Shi Zhaoji (Alfred Sao-ke Sze, 1877–1958), recommended Dr. Wu Lien-teh, a Cambridge-trained physician, as the head of a medical team assigned to help fight the epidemics. Three days after his arrival in Fujiadian, a satellite town (inhabited mainly by the Chinese) within walking distance of the Russian-dominated city of Harbin, he dissected the first corpse of a plague victim and analyzed bacterial cultures from organ specimens (Lei 2014: 25). It was soon clear to him that the organism under his microscope appeared to be identical with the Bacillus pestis that Kitasato Shibasaburo (1853- 1931) and Alexandre Yersin (1863–1943) had co-discovered during the Hong Kong plague in 1894. But Wu observed a novel fact, namely that these bacilli were found exclusively in the victim’s lungs, which could suggest that the plague this time was very different in nature from the one 17 years before. Wu then proposed a “bold theory” (Lynteris 2018: 444) that the plague in Manchuria had taken a pneumonic rather than a bubonic form. The latter had been the newly established knowledge about the plague among the international scientific community since the beginning of the third plague pandemic 15 years before. Wu believed that the plague in Manchuria was not transmitted by infected rat fleas but spread directly from person to person through the air. In order to block the spread of this contagious disease, he designed a gauze cotton mask and recommended that it be worn by doctors and paramedical staff who were involved in the containment of the plague (Wu 1926: 397f). Although the design of Wu’s mask resembled recently established surgical masks used in the operation room at the turn of the twentieth century, it was the first time that gauze masks were used in the context of epidemic control (Lynteris 2018: 444).

It would be too rash and too technically deterministic to assume that Wu’s bold theory with a pneumonic plague would automatically lead to the legitimization of using gauze masks. After all, the mode of disease transmission had been contentious since the outbreak of the Manchurian plague. So was the efficacy of facemasks (Farrar 1912: 15f; Wu 1926: 395). What’s more, the use of facemasks was a rather unthinkable strategy in the Chinese history of plague containment. How could a young physician like Wu, whose bold theory was somewhat contrary to the medical knowledge held by many senior foreign epidemiologists, convince Chinese officials to adopt gauze masks in epidemic control? What kinds of institutional, discursive, and strategic conditions needed to be created so that the use of anti-plague masks could become thinkable, feasible, and perhaps even indispensable?

## A Shift in Plague Epistemology: From *chuanran* to Contagion

The idea of facial masking as prophylactic measures against epidemics had been rather unthinkable to medical practitioners in the Chinese dynasty times. This was primarily due to the fact that contagion was almost never invoked as the cause of epidemics as in the Western tradition (Benedict 1996: 105; Leung 2010: 25). If contagion was mentioned, it tended to be understood as caused by a multitude of factors including for example the *diqi* (earth qi), moral degradation, or bad fengshui rather than by a single cause. Common responses to epidemics in Qing dynasty were “charitable relief, cleanup campaigns, appeals to the plague gods, and participation in community ceremonies” (Benedict 1996: 128). In this sense, the emergence of anti-plague masks in the context of the Manchurian plague articulated a shift in plague epistemology. Central to this shift was a new understanding of contagion informed by germ theory.

The Manchurian plague was indeed of a very special kind. Examining the uniqueness of this epidemic event, Lei draws our attention to an official report submitted to the Qing court after the Manchurian plague in 1911, in which Xi Liang (1853–1917), governor of the region recalled: “In the beginning [of the outbreak], [we] did not believe that this plague could chuanran [spread by contagion or infection]; all the protective and therapeutic measures were based on the conventional ways that China used to cope with wenyi [warm epidemics].” (cite in accordance to the original version in Lei 2010, 94).

The Chinese term *chuanran* was commonly used by medical practitioners to describe the transmission of diseases after the twelfth century (Leung 2010, 37). It is better to locate traditional understandings of chuanran in what Rosenberg called the configuration approach (Kuriyama 2000: 13), in which outbreaks of diseases are considered to be triggered by “a dynamically interacting web of influences” (Leung 2010: 44) in the environment.

In the latter half of the nineteenth century, the word *chuanran* acquired a new meaning as new concepts from biomedicine were translated into the Chinese context. Despite the immeasurability between *chuanran* in the traditional Chinese medical sense and contagion informed by the germ theory, the former was arbitrarily used as the Chinese term to translate the latter (Leung 2010: 45). One should, however, not claim that *chuanran* has since then been used interchangeably with contagion, but rather reserve a more nuanced understanding of *chuanran* that encompassed multiple, mixed, and sometimes ambiguous meanings at the beginning of the twentieth century.

When Xi Liang confessed that local gentry and medical practitioners did not believe that the plague could *chuanran*, he “might already have incorporated modern biomedical ideas of germs” (Leung 2010: 45). With his remark on *chuanran*, Xi performed what Lydia Liu terms as “translingual practice” (Liu 1996), a discursive practice that transgressed the irreducible differences between a multi-causal mode of *chuanran* and a mono-causal one. By doing this, Xi dissolved the ambiguity between the two and justified the latter as the legitimate meaning of *chuanran*. This new understanding of *chuanran* marked a shift in the attitudes of Qing officials towards a new way of understanding and governing epidemics.

In retrospect, Lei (2014, 23) emphasizes the role of germ theory in framing the Manchurian plague 1910/11. The germ theory was introduced into China through the translation of foreign biomedical books and became dominant around 1900 (Leung 2010, 45). It legitimized a series of new anti-plague strategies including the use of masks during the plague in Manchuria. Some of these new measures such as forced quarantine and mass cremation were enforced so rigorously as to be perceived by Manchurian people as “the most brutal policies seen in four thousand years” (Lei 2010: 82). However, the germ theory did not just ‘come’ through translation. As will be shown below, historical anecdotes during the Manchurian plague also played a central role in introducing the idea of deadly viruses to the general public and convincing them of the efficacy of mask-wearing.

## A Decisive Anecdote

As already mentioned, Wu’s diagnosis of an airborne plague was a rather contentious theory at the beginning of the outbreak. Visualizing the plague bacillus, Wu’s microscope helped to convince local officials of the cause of the plague, but the static microscopic image could not show how the pathogen was transmitted (Lynteris 2016: 10). Confident of their updated knowledge about the bubonic plague, many foreign experts ridiculed Wu’s airborne thesis and therefore refused to wear masks even when they were in close contact with plague patients (Lei 2010: 79). Dr. Gérald Mesny, a senior colleague on the Chinese anti-plague team and the head professor of Beiyang Medical College, was one of them. He expressed strong resistance to Wu’s discovery. In his autobiography *Plague Fighter*, Wu recalled their encounter in the third person:

Dr. Wu was seated in a large padded armchair, trying to smile away their differences. The Frenchman was excited, and kept on walking to and fro in the heated room. Suddenly, unable to contain himself any longer, he faced Dr. Wu, raised both his arms in a threatening manner, and with bulging eyes cried out ‘You, you Chinaman, how dare you laugh at me and contradict your superior?’ (Wu 1959: 19)

A few days later, the news arrived that Mesny had become infected with the plague when he visited a Russian epidemic hospital without wearing a mask, and he passed away a few days later. The death of Mesny, a leading figure on the anti-plague team, soon led to a wave of panic in Manchuria (Lei 2010: 80) and constituted “the turning-point” (Nathan 1967: 11) of the anti-plague campaign by the local government. Wu (1959: 22) wrote in his biography later that “[from now on] almost everyone in the streets was seen to wear one form of mask or another”.

The Mesny anecdote can be read as a historical contingency, after all, he might not have died if the plague had not been airborne. On the other hand, it would be anthropocentric to tag everything that is caused by forces beyond human beings as contingent without seriously considering the power of nonhuman actants. This is especially true given that the mortality rate of the Manchurian plague was 100%. The death of Mesny demonstrated what the microscopic lens could not prove: the pneumonic character of the plague in Manchuria (Lei 2010: 80). In this sense, the Mesny anecdote can be read as a central heterogeneous event that legitimized Wu’s monocausal ‘airborne theory’ as well as the necessity to use gauze masks as protective equipment against contagion.

The interest in Wu’s gauze masks was not given at the beginning, nor were they generated through scientific reasoning. The adaptation of anti-plague masks would be impossible without the ‘ally’ of the ferocious virus. In this anecdote, the plague bacillus helped convince the efficacy of facemasks and therefore *transform* them into protective devices, while the latter in turn ‘proved’ the contagious nature of the former. In this sense, one cannot examine the facemask as an isolated object but rather as a “hybrid actor”^1^ (Latour 1994: 33). In other words, the power of masks as personal protective equipment does not emerge from the mask itself, but rather in their assemblages with other elements such as a biomedical understanding of the virus, human bodies, and affects (Lupton et al. 2021: 7–9).

## A Mythic Origin

In a chapter called ‘History of the Mask’ in *A Treatise on Pneumonic Plague*, Wu (1926: 391) revealed a far-reaching connection between anti-plague masks used in the Manchurian plague and plague doctor masks from the seventeenth-century Europe.

Before discussing the measures as practiced to-day [sic], we may dwell shortly upon the various means adopted for personal prophylaxis as practiced in the past. History takes us back to the time of the Black Death, when medical experts were apparently aware that, in the pneumonic type of plague, the infection was directly transmitted through the air. This seems highly advanced by the side of the practices adopted in later centuries, such as in Milan (1630), Rome (1636), etc., when the mask and other prophylactic implements were used without any clear idea of respiratory mode of infection beyond a vague hypothesis of some ‘miasma’ being present.

Putting aside the validity question of Wu’s retrospective diagnosis of the Black Death, his eagerness to brand the gauze mask as the climax of continual progress of personal plague prophylaxis appears problematic today. Wu’s praise of the beaked plague masks used in seventeenth century Europe as “highly advanced” (Wu 1926: 391) seems to be exaggerated.

In a recent study on historical plague doctor masks, Ruisinger (2020: 247) argues that the beaked mask was at best a marginal phenomenon of the plague before the seventeenth century. Ironically, the motif of the plague doctor costume was popularized in the eighteenth century by broadsheets that made fun of the superstitious foreign doctors from Southern Europe. In other words, the plague doctors with their beaked costumes became the symbol of plague par excellence not through their real existence but through the propagandist broadsheets spread around 1700 in Europe.

After all, Wu is not a historian, his misinterpretation of the historical plague mask, however, seems to fit better in his political agenda to introduce Western medicine into China (Lee et al. 2014). As will be demonstrated in the next part, Wu’s eagerness to modernize Chinese medicine and public health was expressed through the visuality of anti-plague masks presented in the first International Plague Conference.

## Biopolitics of Facemasks

By the end of March 1911, new plague cases had begun to decline throughout. One month later, the first International Plague Conference took place in Mukden, a Manchurian city with mainly Chinese residents. Held only months before the collapse of the Qing dynasty, the conference was the first international medical conference in modern Chinese history (Gamsa 2006: 153). It was held to debate the epidemiological and public health aspects of the outbreak. At the same time, the competition among political interests of Russia, China, and Japan over Manchuria culminated.

In the Mukden conference, Wu presented international delegates with a carefully crafted photographic album, in which “the white contour of the mask creates a strong contrast that renders Wu’s anti-epidemic army all the more visible.” (Lynteris 2018: 446) It is interesting to note that 17 years ago, a similar visual strategy based on the color white was also deployed in the internationally circulated photographic documentation of the plague in Hong Kong (1894) (Peckham 2016a: 51). The photographs of ‘Whitewash Brigade’ for example mobilized the trope of ‘white’ to whitewash the ‘backward’ Chinese, as white-uniformed personnel of the ‘Whitewash Brigade’ was authorized to demolish “unsanitary Chinese dwellings in the Western District [in Hong Kong]” (Peckham 2016a: 42). ‘Whiteness’ was used as a trope of cleanness and purity, which contrasts with dirt and danger. The visual contrast between white-uniformed British soldiers and dark shanty plague-affected towns in Hong Kong not only highlighted the ‘backward’ Chineseness as responsible for the transmission of the plague but also visualized the coming of hygienic modernity as a force of purification.

It can therefore be argued that, with the photographic album presented in the Mukden conference, Wu was not simply propagating the medical efficacy of these face-worn apparatuses in containing the plague but also their ‘modernizing' efficacy—their potentiality to transform wearers into subjects of hygienic modernity. The strategic presentation of masks helped Wu to reverse the "civilizational and racial war against the supposedly irreducible link between germs and Chinese backwardness” (Lynteris 2018: 447), and demonstrated that the Chinese were now able to contain the plague with modern biomedical knowledge.

Gauze masks symbolized the ‘dream’ of Chinese medical elites to transform Chinese citizens into medically rationalized subjects. It is, however, hard to estimate the extent to which the medical rationality of mask-wearing was adopted by the general public during the Manchurian Plague. To this issue, Wu (1926: 339) once commented:

We remember seeing a man in the street with a stiff (wire-framed) mask over the nose only, while he was serenely smoking a cigarette. Sometimes the masks were wrapped around the neck ‘as a protection against cold’!

Another example is that some ‘coolies’ stamped the facemask with temple seals before wearing them. This act of stamping was supposed to turn facemasks into amulets (Farrar 1912: 3) These examples show not only how the scientific sense of mask-wearing failed to reach the population, but it also reveals the failure of the Chinese modernizers’ dream to transform its population into medically-reasoned subjects.

## A Shift of Perspective: SARS and the Popularization of Mask-wearing

Different models (of masks) were worn during the first Manchurian epidemic […] Only one kind of mask, at once simple and inexpensive, yet apparently efficient, introduced by Wu Lien-Teh, was universally adopted. (Wu 1926: 393)

In the previous section, the politics of mask-wearing has been primarily interrogated from a ‘macro’ perspective that focuses on medical, historical, and political discursive entanglements immanent in the emergence of anti-plague masks. In this part, the analytical attention will be shifted to a ‘micro’ perspective that gives emphasis to the formation of a particular ‘pro-mask subjectivity’. The reason for such a shift of perspective has to do with the historical development of facemasks: During the 1918 influenza and especially in its more recent history in East Asia since 2000, the facemask has become a more popularized and individualized device of anti-contagion prophylaxis. The popularization of facemasks since SARS was accompanied largely by disposable surgical masks that progressively replaced the reusable cotton masks with the rise of throwaway culture from the 1960s (Strasser and Schlich 2020). Made of non-woven synthetic fibers, they were widely adopted because they are relatively inexpensive, convenient, and efficient. The ‘economy’ of surgical masks not only gave rise to a quantitative proliferation of masking practices but also qualitatively to new social meanings. During the SARS outbreak in 2002/3, surgical masks became a commodity that was actively purchased and, more importantly, a marker of care and solidarity in the zeitgeist of neoliberalism.

While during the Manchurian plague, the use of gauze masks was rather initiated by medical authorities, in the wake of the twenty-first century, the situation was almost reversed: The general public in China was willing to wear facemasks during the SARS outbreak, even before masks were made obligatory. In what follows, this article attempts to handle these questions: Despite the controversy over the efficacy of facemasks, which factor gave rise to the seemingly ‘voluntary’, ‘dutiful’, and collective use of facemasks during the SARS epidemic? In which way were people incited to recognize their obligations to wear facemasks? What was the telos of this collective masking practice? What was the most salient experience during this time?

## SARS and Ubiquitous Facemasks

In November 2002 in south China, Severe Acute Respiratory Syndrome (SARS) broke out and rapidly swept across the globe. The first documented case of SARS was identified in Foshan, Guangdong province on 16 November 2002. In February 2003, SARS moved beyond the Chinese mainland when a doctor (regarded by the WHO as the ‘super spreader’) from Guangdong who had treated SARS patients stayed in a hotel in Hong Kong (Peckham 2016b: 285). The threat of SARS was often associated with its rapid spread and its ‘global reach’. By July 2003, it had affected over 8000 people worldwide (mainly in South East Asia and North America), leaving at least 774 dead (Duffin and Sweetman 2010: 1).

During its outbreak, SARS was framed by WHO as a “novel” disease that posed “a particularly serious threat to international health” (WHO 2003). Although the last decades of the twentieth century had witnessed the emergence of several new diseases, SARS was seen as “the first severe infectious disease to emerge in the globalized society of the twenty-first century” (Tseng and Wu 2010: 260). This is primarily due to its novel pathogenicity, easy transmissibility, and a globalized infrastructure that facilitates its rapid spread. Interestingly, despite an early recognition of the novelty of the SARS disease through an international collaboration that identified its genetic sequence as a new strain of coronavirus, public health measures to contain SARS remained antiquated and uncreative. Summarizing the lessons learned from 2003, the WHO drew attention to the low-tech containment strategies adopted:

While modern science had its role, none of the most modern technical tools had an important role in controlling SARS. Sequencing the genetic code of the virus, for example, helped identify the origin and spread of the virus but did not really help to control it [...] Most important in controlling SARS were the 19th-century public strategies of contact tracing, quarantine, and isolation. (WHO 2006: 247)

This remark also applies to the strategy of masking which emerged during the Manchurian plague 1910–11. Interestingly, similar to this plague event, the efficacy of mask-wearing was also a matter of controversy among international scientific communities during the SARS outbreak. In China, the initial stance at the beginning of the outbreak was even to “ridicule the use of facemasks” (Sin 2016: 91). According to Dr. Lo Wing-lok, an authority in the field, he questioned the effectiveness of mask-wearing, commenting that “as the tiny holes in a mask cannot filter out the viruses in the air, one can also breathe them in and get infected”. Following this remark, the Next Magazine then criticized an ‘unscientific’ public manner towards masking-wearing that “everyone in the hospital, even the cashier behind the glass counter and the cleaning workers were wearing a mask or several masks”. (Next Magazine 2003, cited in Sin 2016: 91).

Although the expert knowledge questioned the effectiveness of facemasks ‘from above’, there seemed to be a kind of ‘voluntariness’ or ‘autonomy’ among the public when it comes to mask-wearing. Doubts over the efficacy of facemasks did not stop people from wearing them or purchasing them in bulk. Many Chinese started to wear improvised protective gears on the street, and this creativity was also reported by the media. Subsequently, the official stance toward facemasks changed, however.

As fear escalated, the state media began to press authorities to ensure a mask-wearing behavior in the population. Masks became ubiquitous. Of all the articles that contain the keyword SARS during the sampled period, 80% also mention masks. (Sin 2016: 91)

It would be incorrect to say that facemasks became ubiquitous in all of China during the SARS outbreak, but at least, this was true in the SARS-affected areas and cities such as Guangzhou, Beijing, and Hong Kong. Another important observation was that before 2003, facemasks had rarely been seen in public areas in Hong Kong and mainland China, so the emergence of everyday masking practice was almost a rupture. Following Burgess and Horii (2012)’s insights into the popularization of masking practice in Japan, this article argues that to understand the similar process in the Chinese SARS context, it is vital to examine the formation of responsibilized subjects before the SARS outbreak. The widespread use of facemasks can be seen as an effect of the neoliberal strategy deployed since the 1980s.

## Detour: Responsibilized Citizens and Mask-wearing

Before discussing the formation of responsibilized subjects as a key cornerstone of the widspread mask-wearing practices in the Chinese context, this article will first turn to a closely related but complex question. It can be argued that the production of responsibilized subjects is a general strategy of neoliberal governmentality that can be observed in both Western and East Asian societies since the 1980s. Now the question is: Why do some responsibilized subjects formed in one context tend to have a higher acceptance towards facemasks than their counterparts in another context?

First, the popularization of facemasks among a population and the seemingly wide acceptance of them is rather a historical contingency induced by a combination of forces from the mass media, business interests, and political orderings, for example. Many European countries were relatively less hit by the recent pandemics such as SARS or H1N1. These events gave rise to collective masking strategies in East Asia, South, and North America.

Second, the so-called ‘mask culture’ (Zhang 2021) can be understood as an effect of neoliberal strategies so far as the formation of responsibilized subjects is specified with context-specific terms. This includes taking into account various cultural perceptions of face-covering practices, cultural-specific understandings of disease and risk, and different risk management strategies. For example, examining the Japanese language uses that express germs, dirt, and influenza, Burgess and Horii (2012, 1184) argued that the practice of mask-wearing “resonated with folk assumptions as making a barrier between purity and pollution”. At the same time, they were careful not to make any culturally essentialist claims as if there were a fixed cultural embracement of facemasks. Nevertheless, this specific cultural understanding of disease and risk intersects with the process of responsibilization of individuals in Japan since the 1990s, thus creating a specific subjectivity with a sense of individuated insecurity.

In the Chinese context, it can be argued that the sudden widespread practice of mask-wearing during the SARS outbreak is a result of complex social and political processes, including e.g. the overexposure of facemasks in the media, expert advising, the enforcement of mask-wearing through law and regulations, political orderings, and newly formed social norms that favor mask-wearing. However, they do not constitute the most salient factor here. After all, many people were wearing masks during the SARS outbreak before all these ‘social determinants’ could ‘structure’ their behavior. To understand this proactive practice, it is vital to shift attention to their ethical world and to explore their “mode of subjection” (“mode d’assujettissement”) namely, “the way in which people are invited or incited to recognize their moral obligations” (Foucault 1984, 354).

## Self-cultivating Subjects: Mask-wearing and Care

Since China’s economic reform policies in 1978, its health system has experienced a similar process of liberalization and privatization. The previous fully state-financed health care system was shifted towards a more commercialized and privately funded one, which was accompanied by soaring medical fees, poor access to affordable medical services, and a decline of medical insurance coverage (Tu 2019: xxi). This process, though interlinked with the contemporary structural change of economic liberation, has also been embedded in ‘traditional’ Confucian ethics concerning the self. In this section, the focus will be on the Confucian practice of *xiushen* (self-cultivation) and its role in the formation of responsibilized ethical subjects in the course of commercialization and privatization of the Chinese health care system.

In the collective era between 1949 and 1976, self-cultivation was integrated into a new discourse of ‘*zili gengsheng*’ (self-reliance), propagated under Mao’s leadership that Chinese people could survive and thrive by their own efforts. Although in cities, especially for those who belonged to a *danwei* (work unit), health care was largely understood to be the responsibility of the state or the collective, the majority of the population, especially those in the rural areas, were outside the *danwei* system (Tu 2019: 65). Since the 1980s, with the gradual dissolution of the *danwei* system, the health care burden was further shifted from the state to individuals. At the same time, health care activities became increasingly linked with consumption activities. The marketization of the health care system slowly gave rise to a new self-caring subjectivity that had to learn to negotiate in the market economy. As a result, individuals are faced with increasing uncertainties over medical charges and are encouraged to become ‘calculative’ (Tu 2019: 68f). The goal for the neoliberal self-responsible subjects is to consume the right products, which “has increasingly become a technique of self-management.” (Tu 2019: 72) One must be careful that the health care responsibility is not shifted to an independent self with a clear boundary from the other, but rather an “interdependent self-defined by one’s social role and relationships” (Hwang 1998: 21f).

Callahan (2006: 14) noticed “interesting parallels between Confucianism and the pastoral politics of governmentality": A neo-Confucianist understanding of governance “transgresses the binary distinctions of state and civil society to make individuals, civil society and state conterminous and mutually entailing.” According to Daxue (The Great Learning), one of the Four Books of the Neo-Confucian orthodoxy, self-cultivation is rarely a self-indulgent and narcissistic practice but rather a departure point for the proper order of family and state. In this sense, there is a striking similarity between the Confucian self-cultivation and the Foucauldian ‘care of the self’ (Hahm 2001).

Confucian ethics such as *xiushen* played a central role in ‘appealing’ citizens towards self-cultivating subjects, which constitute the cornerstone of the often taken-for-granted ‘mask culture’ in China. Mask-wearing can be seen not only as an act of self-cultivation but also as the Foucauldian ‘care of the self’ that involves “knowing and fulfilling one’s duties toward others, including one’s spouse, children … and fellow citizens.” (White 2014: 498). In the Chinese context, the paradigmatic process of individualization and liberalization since the 1980s, to put it schematically, did not give rise to subjects who perceive the facemask as limiting their freedom, but rather those who perceive it as an embodiment of care. During the SARS outbreak, facemasks embodied the ‘right’ health product and a ‘rational’ choice in the health care market, especially when no medical cure for the SARS disease was available. People purchased facemasks, scrambled for them, hoarded them, and at the same time, facemasks were given as personal gifts or as the ‘care’ of the company (Lynteris 2013: 175). Facemasks involve not only self-care but also care for others. It is a kind of coterminous understanding of the care that care of the self, care for family members, civic duty (care for the state), and vice versa, being cared for by the state, and being cared for by the family are mutually entailing. Although Foucault’s ‘care of the self’ is primarily based on his examination of historical texts from ancient Greece and the later Hellenistic/Roman period, this concept can be useful for conceptualizing mask-wearing in that it points to an ethical question of relation to oneself and relation to others, and of the entanglement of the two.

## A salient Masking Experience: Mask-wearing as Risk Ritual

SARS can be understood epidemiologically as a viral epidemic that tested Hong Kong’s healthcare system but also symbolically as an epidemic of fear testing the city’s moral existence (Baehr 2008). Even though SARS in Hong Kong ended up with a death toll of ‘only’ 299 people, this knowledge was not available at its outbreak (Baehr 2008: 139). SARS brought about mass insecurity and panic in Hong Kong, with its sudden appearance, its unique pathogen for which no standard medical cure was available, and its puzzling mechanism of transmission. To make things worse, in Hong Kong, officials were reluctant to use the term SARS, choosing instead to call it ‘atypical pneumonia’ (Peckham 2016b: 284f). In both the Chinese and English language media, martial terminology and disease-as-war imagery were pervasive (Baehr 2005: 148). The medical staff was described as ‘frontline’ workers or as ‘troops’ fighting against the virus. Other words such as ‘battleground’, ‘hero’, or ‘sacrifice’ were also suddenly ubiquitously used by media and citizens. Meanwhile, the withholding and censoring of information during SARS by the central government in Beijing led to a massive distrust of official information, which further aggravated misinformation about the disease and the infectious circulation of panic (Rojas 2015: 160f). It is against this backdrop of drastic mental experience of panic, massive public emotional charge, and collective disempowerment, that Baehr (2008: 150) observed that Hong Kong has suddenly turned into a ‘masked city’:

mask-wearing became the quickly improvised, if obligatory, social ritual; failing to don one was met with righteous indignation, a clear sign of ritual violation. The mask symbolized a rule of conduct – namely, an obligation to protect the wider community – and an expectation regarding how one was to be treated by others. More simply, the mask was the emblematic means by which people communicated their responsibilities to the social group of which they were members.

Interestingly, instead of transforming its wearers into scientific-minded and composed subjects who ‘valiantly’ participated in the ‘war’ against the virus, mask-wearing became a social practice of “risk ritual” (Moore and Burgess 2011). Different from religious and community rituals such as rain dance or religious ablutions, risk rituals such as masking practice or repeated hand-washing due to epidemic uncertainty should be “better viewed as [symbolically] functional rather than irrational” (Moore and Burgess 2011: 111). Regardless of its practical effects, the symbolic role of ritualistic risk practice is to dissipate or to replace feelings of uncertainty, affirming group norms and their continuation (Moore and Burgess 2011: 119).

During the SARS outbreak in Hong Kong, mask-wearing became ritualistic the moment when its practical efficacy was secondary to its symbolic role of lessening uncertainty and anxiety. However, mask-wearing in response to SARS in Hong Kong was a risk ritual “only in the most general sense of the term” (Burgess and Horii 2012: 1194). Since its efficacy has been scientifically contested, the significance of mask-wearing was “not yet latent” (Burgess and Horii 2012: 1194). Unlike hand-washing, mask-wearing as a risk ritual is unstable and tends to perpetuate anxiety.

## Conclusion

This article has conducted a genealogical study of facemasks and has focused on the emergence of two differing masking strategies in China’s modern history of epidemic control. The first moment of emergence concerns the birth of modern facemasks, whose design and form remained almost unchanged till today. They were more than medical masks. The rationalities of implementing them intermingled with strategical socio-political concerns of late-Qing reformers at the crossroad of historical transformation. Around 90 years later, in another epidemic event 2002/3, a new facemask regime emerged, whose subjects themselves are willing to wear masks and give meaning to this act. The economy of facemasks has come to play a dominant role. The proliferation of disposable and inexpensive surgical masks paved the way for the sudden appearance of a universal masking strategy during the SARS epidemic. The mask economy not only helped a liberal government to shift health care responsibility to the public but also gave rise to an ethical problem. Mask wearing became a sign of care, yet in some cases, it became a risk ritual in order to protect the wider community. The so-called community mask can be seen as a product of liberal biopolitics of masking strategy.

A genealogical study of facemasks is not just a historical exploration of masking; the aim is rather to rethink facemasks in the present. First, they should not be treated as inanimate objects ‘out there’, but actants who actively participate in the constitution of the world we share. Masks “alone do not posseses or exert agency: it is only in their assemblages with other actors that agency is generated and expressed” (Lupton et al. 2021: 7). Masks communicate danger, evoke fear, provide a sense of security, affect communication, impede breathing or impair freedom. In terms of subject formation, they inform and transform our identity. The act of mask-*wearing* is more than donning a piece of cloth upon one’s face, it is also a matter of an ethical concern of self-care and care for others. In some cases, to mask or not become a political issue (Lupton et al. 2021: 20–23). Existing social and political divides were articulated and reproduced through this piece of face-covering fabric (Ma and Zhan 2020).

Second, this article argues for a rethinking of facemasks in terms of multiplicity.^2^ This does not only refer to their various designs in terms of materiality, comfortability, and efficiency but also, and especially, to their multiple functionalities. Face-worn protective devices come to appear in different contexts different forms: medical masks, community masks, masks that are seen as a commodity, and those that are worn as a political statement. This does not mean shifting the attention to the pluralism of different versions of masks, but rather to a more situated and context-sensitive approach that reconstructs how they relate.

## Data Availability

Not applicable.

## Footnotes

1 Hybrid actors are illustrated by Latour (1994: 33) with the citizen-gun example, in which both the citizen and the gun are transformed into another subject: a good citizen becomes a criminal and a silent gun becomes a weapon. This concept rejects an essentialized reading of human and nonhuman entities as if they could be analyzed separately. Instead, the focus of analysis is shifted to the co-influence among heterogeneous actants and their configurations.

2 Mol’s ‘ontological multiplicity’ invites us to regard reality in terms of multiplicity instead of singularity. This is not because reality is seen from different perspectives, but rather it is enacted in practices in multiple ways. In Mol’s ethnographic study of atherosclerosis, she argues that atherosclerosis does not exist a priori, but comes into being through a multitude of medical practices that produce multiple forms of the’same’ disease. Nevertheless, multiplicity is not about a plurality of things, but a coexistence of multiple forms of the same thing at the same time. “[F]ar from necessarily falling into fragments, multiple objects tend to hang together somehow.” (Mol 2003: 5).
